# Post-transcriptional regulatory network of epithelial-to-mesenchymal and mesenchymal-to-epithelial transitions

**DOI:** 10.1186/1756-8722-7-19

**Published:** 2014-03-05

**Authors:** Fei Guo, Brittany C Parker Kerrigan, Da Yang, Limei Hu, Ilya Shmulevich, Anil K Sood, Fengxia Xue, Wei Zhang

**Affiliations:** 1 Department of Pathology, Unit 85, The University of Texas MD Anderson Cancer Center, 1515 Holcombe Blvd, Houston, TX 77030, USA; 2 Gynecologic Oncology and Reproductive Medicine, The University of Texas MD Anderson Cancer Center, 1515 Holcombe Blvd, Houston, TX 77030, USA; 3 Cancer Biology, The University of Texas MD Anderson Cancer Center, 1515 Holcombe Blvd, Houston, TX 77030, USA; 4 Center for RNAi and Non-Coding RNA, The University of Texas MD Anderson Cancer Center, Houston, Texas, USA; 5 Department of Gynecology and Obstetrics, Tianjin Medical University General Hospital, No. 154, Anshan Rd, Heping District, Tianjin 300052, People’s Republic of China; 6 Institute for Systems Biology, Seattle, Washington, USA

**Keywords:** Long non-coding RNA (lncRNA), microRNA (miRNA), Epithelial-to-mesenchymal transition (EMT), Mesenchymal-to-epithelial transition (MET)

## Abstract

Epithelial-to-mesenchymal transition (EMT) and its reverse process, mesenchymal-to-epithelial transition (MET), play important roles in embryogenesis, stem cell biology, and cancer progression. EMT can be regulated by many signaling pathways and regulatory transcriptional networks. Furthermore, post-transcriptional regulatory networks regulate EMT; these networks include the long non-coding RNA (lncRNA) and microRNA (miRNA) families. Specifically, the miR-200 family, miR-101, miR-506, and several lncRNAs have been found to regulate EMT. Recent studies have illustrated that several lncRNAs are overexpressed in various cancers and that they can promote tumor metastasis by inducing EMT. MiRNA controls EMT by regulating EMT transcription factors or other EMT regulators, suggesting that lncRNAs and miRNA are novel therapeutic targets for the treatment of cancer. Further efforts have shown that non-coding-mediated EMT regulation is closely associated with epigenetic regulation through promoter methylation (e.g., miR-200 or miR-506) and protein regulation (e.g., SET8 via miR-502). The formation of gene fusions has also been found to promote EMT in prostate cancer. In this review, we discuss the post-transcriptional regulatory network that is involved in EMT and MET and how targeting EMT and MET may provide effective therapeutics for human disease.

## Introduction

EMT is a process whereby epithelial cells lose both polarity and cell-to-cell contacts. Cells undergoing EMT acquire a mesenchymal phenotype, which is characterized by an epithelial-to-mesenchymal switch in marker expression, such as the loss of epithelial markers (e.g., E-cadherin, claudin, and occludin) and gain of mesenchymal markers (e.g., vimentin and N-cadherin). The reverse process, known as mesenchymal-to-epithelial transition (MET), has also been reported [1]. EMT and MET are important in organ development, stem cell biology, wound healing, and cancer progression. Many signals, transcriptional factors, and post-transcriptional regulatory networks can induce EMT. Post-transcriptional regulatory networks include the miRNA and lncRNA families. Therefore, in this review, we focus on miRNA and lncRNA, which may be effective diagnostic and therapeutic targets in cancer. Specifically, we describe several lncRNAs that regulate EMT in cancer, as well as miRNAs that regulate multiple signaling pathways involved in EMT and transcription factors of E-cadherin.

### EMT and MET regulate important processes, including disease

EMT and MET have central roles in embryogenesis and cancer metastasis [2]. EMT is an integral part of tissue remodeling that occurs during embryogenesis [1]. MET also contributes to embryonic development [3]. In adults, EMT can be activated to promote wound healing after tissue injury [4]. EMT induction allows cancer cells to disseminate from the primary tumor, invade surrounding tissues, and eventually generate metastases by colonizing remote sites via blood or lymphatic routes. Metastatic cells can then revert back via MET to re-acquire epithelial characteristics similar to those of cells in the primary tumor [4].

EMT and MET are essential to the regulation of stem cell pluripotency [4]. Tumors contain cancer stem cells (CSCs), which are a small subpopulation of cells that are capable of self-renewal, differentiation, and tumorigenicity. Evidence suggests that EMT induction enhances self-renewal and the acquisition of CSC characteristics [5]. Thus, therapeutics that target EMT may be useful for reducing CSC populations in cancer.

### Key regulators involved in EMT and MET

During EMT, epithelial cells lose cell-to-cell interactions, undergo morphological challenges, and increase their cellular motility. The most important mediator of cell-to-cell adhesion is the cadherin family of proteins, which promotes the formation of adherens junctions that act as glue to hold the cells within tissues together. The most characterized cadherins include E-, N-, and P-cadherin. E-cadherin plays an important role in epithelial cell-to-cell interactions because it is responsible for holding neighboring epithelial cells together in a classic cobblestone structure. During EMT, E-cadherin is replaced by abnormal expression of N- or P-cadherin. The downregulation of E-cadherin leads to the release of β-catenin, and the latter translocates to the nucleus and functions as an activator for transcription factors, promoting cellular adhesion, tissue morphogenesis, and cancer development.

**Figure 1:**
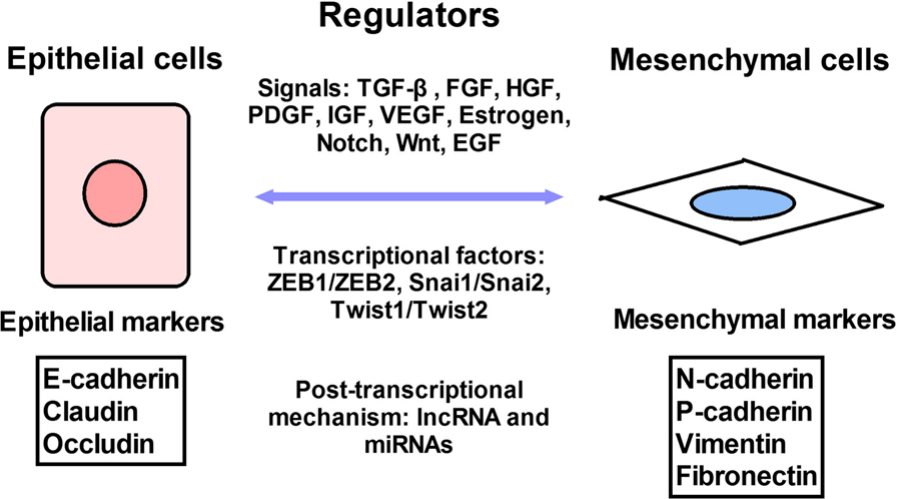
**Regulatory network in EMT and MET.** EMT can be regulated by many signaling pathways, transcription factors, and post-transcriptional mechanisms.

Other proteins that mediate EMT include vimentin and fibronectin. Vimentin is an intermediate filament protein that is upregulated in cells undergoing EMT. During EMT, vimentin expression causes epithelial cells to acquire a mesenchymal shape and increased motility [6]. Fibronectin mediates cellular interactions with the extracellular matrix and is important for migration, differentiation, growth, and cell adhesion. Like vimentin, fibronectin is also upregulated during EMT and can therefore be used as a biomarker for EMT (Figure 1).

EMT is regulated by many signaling pathways, transcriptional factors, and post-transcriptional factors. Many signals, including transforming growth factor-β (TGF-β), fibroblast growth factor (FGF), human growth factor (HGF), platelet-derived growth factor (PDGF), insulin-like growth factor (IGF) (and its receptor [IGFR]), vascular endothelial growth factor (VEGF), estrogen receptor (ERα), Notch, Wnt, and epidermal growth factor (EGF) may be involved in EMT. These pathways ultimately activate the transcription of EMT-related transcription factor families, including ZEB (ZEB1 and ZEB2), the zinc finger Snail (SNAI1 and SNAI2), and the basic helix-loop-helix (e.g., Twist1 and Twist2) [7]. By regulating the expression of E-cadherin, these transcription factors dynamically modulate EMT, cell adhesion, and motility. In addition to transcription factors, EMT is regulated by post-transcriptional mechanisms, including lncRNA and miRNA (Figure 1).

### Regulation of EMT by lncRNAs

LncRNA, which is larger than 200 nt, consists of a heterogeneous group of RNA molecules that are involved in a broad spectrum of cellular processes and in cancer progression [8]. Studies have demonstrated that lncRNAs are aberrantly expressed in a variety of human cancers, such as gastric cancer [9,10], bladder cancer [11,12], and breast cancer [13]. Interestingly, a recent report revealed that several lncRNAs may be involved in EMT regulation [14].

Several important lncRNAs are reported to induce EMT, including highly upregulated in liver cancer (HULC), metastasis-associated lung adenocarcinoma transcript 1 (MALAT-1), H19, and HOX transcript antisense intergenic RNA (HOTAIR).

HULC overexpression in gastric cancer was found to be correlated with lymph node metastasis, distant metastasis, and advanced tumor node metastasis stage [9]. Silencing of HULC effectively reversed the EMT phenotype [9]. MALAT-1 expression was remarkably increased in primary tumors that subsequently metastasized compared with those that did not metastasize. MALAT-1 promoted EMT by activating Wnt signaling *in vitro*[11]. H19 enhanced bladder cancer metastasis by associating with EZH2 and inhibiting E-cadherin expression [12].

The expression level of HOTAIR was significantly correlated with lymph node metastasis and TNM stage in gastric cancer. The results of *in vitro* studies suggested that HOTAIR promoted EMT by regulating Snail [10]. HOTAIR remodels the gene expression pattern of breast epithelial cells into a pattern that more closely resembles that of embryonic fibroblasts, leading to increased cancer invasiveness and metastasis [13]. HOTAIR resides in the mammalian HOXC locus and recruits the polycomb repressive complex 2 to specific target genes genome wide, leading to histone H3 lysine 27 trimethylation and epigenetic silencing of metastasis-suppressor genes.

The findings described above indicate that lncRNA has a role in tumor diagnosis and therapy. Recently, the therapeutic potential of targeting MALAT-1 was demonstrated, as free uptake of antisense oligonucleotides that target MALAT-1 in tumors prevented lung metastasis in nude mice [15]. Together, these results suggest that therapy is needed that hinders cancer progression by targeting specific lncRNAs that are implicated in EMT and therefore metastasis.

### Regulation of EMT by miRNA

A second post-transcriptional mechanism that contributes to EMT involves miRNAs, which are 22-nucleotide non-coding RNAs that suppress gene expression through mRNA destabilization or translational inhibition. They are deregulated in a wide variety of human cancers [16] and have been shown to contribute to the control of cell growth, differentiation, and apoptosis, which are important to cancer development and progression [17]. MiRNAs can regulate multiple signaling pathways involved in EMT. Specifically, they can directly target transcription factors of E-cadherin and other EMT regulators.

#### MiRNAs regulate signaling pathways involved in EMT

**Figure 2:**
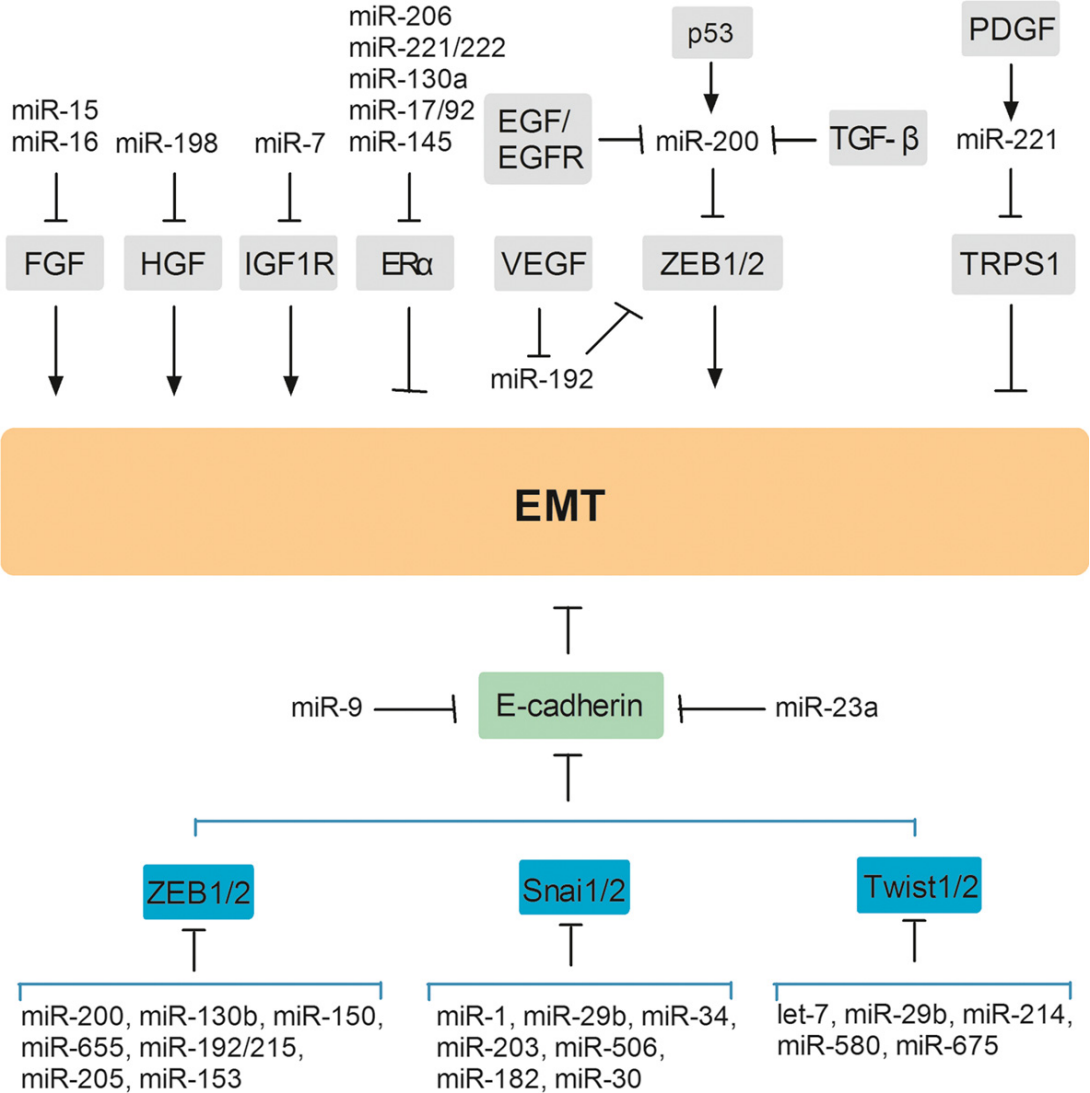
**MiRNA-regulating signaling pathways and transcription factors are involved in EMT.** MiRNA influences EMT by targeting ligands, receptors, signaling pathways and transcription factors.

Many miRNAs influence the EMT process by targeting the expression of specific ligands, receptors, and signaling pathways (Figure 2). Increasing evidence indicates that miRNAs regulate EMT by targeting key EMT regulators, including FGF (and its receptor [FGFR]), HGF, IGF (and IGFR), ERα, Notch, and Wnt. MiR-15 and miR-16 were downregulated in cancer-associated fibroblasts (CAFs) surrounding prostate tumors. This downregulation promoted tumor growth and progression through reduced post-transcriptional repression of FGF-2 and its receptor FGFR1, which act on both stromal and tumor cells to enhance cancer cell survival, proliferation, and migration [18]. Emerging evidence indicates that miR-198 is downregulated in hepatocellular carcinoma compared with in normal liver parenchyma, and forced expression of miR-198 inhibited HGF’s promotion of hepatocellular carcinoma cell migration and invasion in a c-MET-dependent manner [19]. A recent report showed that miR-7 suppresses Snail, increases E-cadherin expression, and partially reverses EMT by targeting IGF1R, generating a novel miR-7/IGF1R/Snail axis in gastric cancer [20]. As we know, ERα signaling opposes EMT by inhibiting TGF-β and cytokine signaling through Smad and nuclear factor-κB. Another report demonstrated that miR-206, miR-221, miR-222, miR-130a, miR-17, miR-92, and miR-145 could suppress ERα and promote EMT [21]. MiR-34a can regulate EMT by directly targeting Notch1 and Jagged1 [22]. Furthermore, a recent study illustrated that miR-200 members can target Jagged1, thereby mediating the downregulation of ZEB1 [23]. The Wnt/β-catenin signal pathway promotes EMT in cancer, and miR-200a was found to inhibit Wnt/β-catenin by targeting ZEB1 and ZEB2. MiR-200 can directly target β-catenin mRNA, inhibiting its translation and blocking Wnt/β-catenin signaling in meningioma [24].

MiRNA can also regulate EMT by targeting signaling pathways, including the TGF-β, PDGF, VEGF, and EGF pathways. TGF-β is a well-known EMT initiator. Exposing epithelial cells to TGF-β promotes the loss of epithelial morphological features, the increased expression of EMT marker genes such as ZEB1 and ZEB2, and the decreased expression of miR-200 [25]. Furthermore, downregulating paracrine TGF-β can inhibit and reverse EMT by downregulating ZEB1 and ZEB2 and upregulating miR-200b and miR-200c [26]. Inhibition of the Smad signaling pathway completely blocked the TGF-β1-mediated decrease in miR-200, suggesting that TGF-β1-induced suppression of the miR-200 family is regulated via Smad [27]. In addition, miR-99a and miR-99b may function as modulators within a complex network of factors that regulate TGF-β-induced EMT [28].

Anping Su et al. demonstrated that downregulation of TRPS1 by miR-221 is critical for the PDGF-mediated EMT phenotype [29]. VEGF was reported to suppress EMT by inhibiting the expression of miR-192 [30], which increases E-cadherin levels via repressed translation of ZEB2 mRNA [31]. Similarly, it was reported that EGF and EGFR can promote EMT by downregulating the miR-200 family in anaplastic thyroid cancer cells [32]. Furthermore, miR-155 overexpression suppressed EGF-induced EMT, decreased migration and invasion, inhibited cell proliferation, and increased chemosensitivity to DDP in human Caski cervical cancer cells [33]. Together, these data underscore the importance of miRNAs in EMT and malignant tumor progression.

### MiRNAs that regulate E-cadherin transcription factors ZEB1 and ZEB2

The expression of E-cadherin is mainly controlled by three families of transcription factors: SNAI1 and SNAI2, ZEB1 and ZEB2, and Twist1 and Twist2. Several miRNAs directly target these families to modulate EMT in cancer (Figure 2). Members of the miR-200 family (miR-200a, miR-200b, miR-200c, miR-141, and miR-429) have emerged as important regulators of EMT, in part by targeting ZEB1 and ZEB2. Moreover, some signaling pathways, including p53, regulate EMT by regulating the miR-200-ZEB1 and ZEB2 regulatory loop.

The miR-200 family is usually downregulated in human cancer cells and tumors as a result of aberrant epigenetic gene silencing. The results of recent studies suggest that members of the miR-200 family play a critical role in suppressing EMT and cancer invasion and metastasis [34] by targeting transcriptional repressors of ZEB1 and ZEB2 [35]. Meanwhile, ZEB1 can directly suppress miRNA-200 family members in cancer cells, including miR-141 and miR-200c [36,37]. It was also reported that ZEB1 and ZEB2 repressed the expression of miR-200a, miR-200b, and miR-429 by binding to a conserved pair of ZEB-type E-box elements located proximal to the transcription start site in the promoter region [38]. Therefore, ZEB1 and ZEB2 and miR-200 family members repress expression of each other in a reciprocal feedback loop, which may lead to amplification of EMT. Targeting this loop may be a new therapeutic strategy for cancer.

#### Pathways that suppress EMT by upregulating miR-200 and repressing ZEB1 and ZEB2

Several molecules have been found to upregulate the miR-200 family and consequently suppress EMT. For example, both P300 and PCAF act as cofactors for ZEB1, forming a P300/PCAF/ZEB1 complex on the miR200c/141 promoter. This results in lysine acetylation of ZEB1 and releases ZEB1’s suppression of miR-200c/141 transcription [39]. Smad3 was also reported to upregulate miR-200 family members at the transcriptional level in a TGF-β-independent manner [40]. p53 has been reported to transactivate miR-200 family members by directly binding to the promoters that repress ZEB1 and ZEB2 expression, leading to inhibition of EMT [41,42]. Similarly, NPV-LDE-225 suppressed EMT by upregulating E-cadherin and inhibited N-cadherin, Snail, Slug, and ZEB1 by increasing miR-200a, miR-200b, and miR-200c [43].

#### Pathways that promote EMT by suppressing miR-200 and upregulating ZEB1 and ZEB2

In addition to their role in regulating EMT, miR-200 family members are negatively regulated by multiple signaling pathways. For example, in one study, overexpression of Stat3 [44], PDGF-D [45], Notch-1 [46], and DCLK1 [47] in cancer cells led to significant downregulation of miR-200 family members; this resulted in up-regulation of ZEB1, ZEB2, and SNAI2 expression and acquisition of the EMT phenotype. IDH1 and IDH2 mutants also caused an EMT-like phenotype; this phenotype was dependent on upregulation of the transcription factor ZEB1 and downregulation of miR-200 family members [48]. Other miRNAs can induce EMT by downregulating miR-200 through DICER, such as miR-103 or miR-107 [49]. Similarly, miR-130b silencing can restore DICER1 to a threshold level that allows miR-200 family members to repress EMT in endometrial cancer [50]. All of these findings indicate that these molecules promote EMT by suppressing miR-200.

Fusion genes are formed when chromosomal instability causes two genes that normally exist in isolation to fuse together [51]. Interestingly, a well-known gene fusion in prostate cancer that is produced by deletion of a 3-mega base region between *ERG* and *TMPRSS2*[52] (also reviewed in [Parker 2014 Journal of Pathology]) has also been found to upregulate ZEB1 and ZEB2 expression [53]. Specifically, expression of the *TMPRSS2-ERG* fusion gene caused epithelial immortalized prostate epithelial cells to undergo morphological changes consistent with those of mesenchymal cells while downregulating expression of the epithelial marker *CDH1*[53]*.* This finding highlights the complex ways in which EMT can be facilitated at the genetic level, simply by the fusion of two genes.

### Epigenetic regulation of miR-200

MiR-200 family members can also be epigenetically regulated. It was reported that miR-200c expression was epigenetically regulated in CRC [54]. Rui Neves et al. also showed that the miR-200c/141 cluster is repressed by DNA methylation of a CpG island located in the promoter region of these miRNAs in invasive breast cancer cells [55].

**Figure 3:**
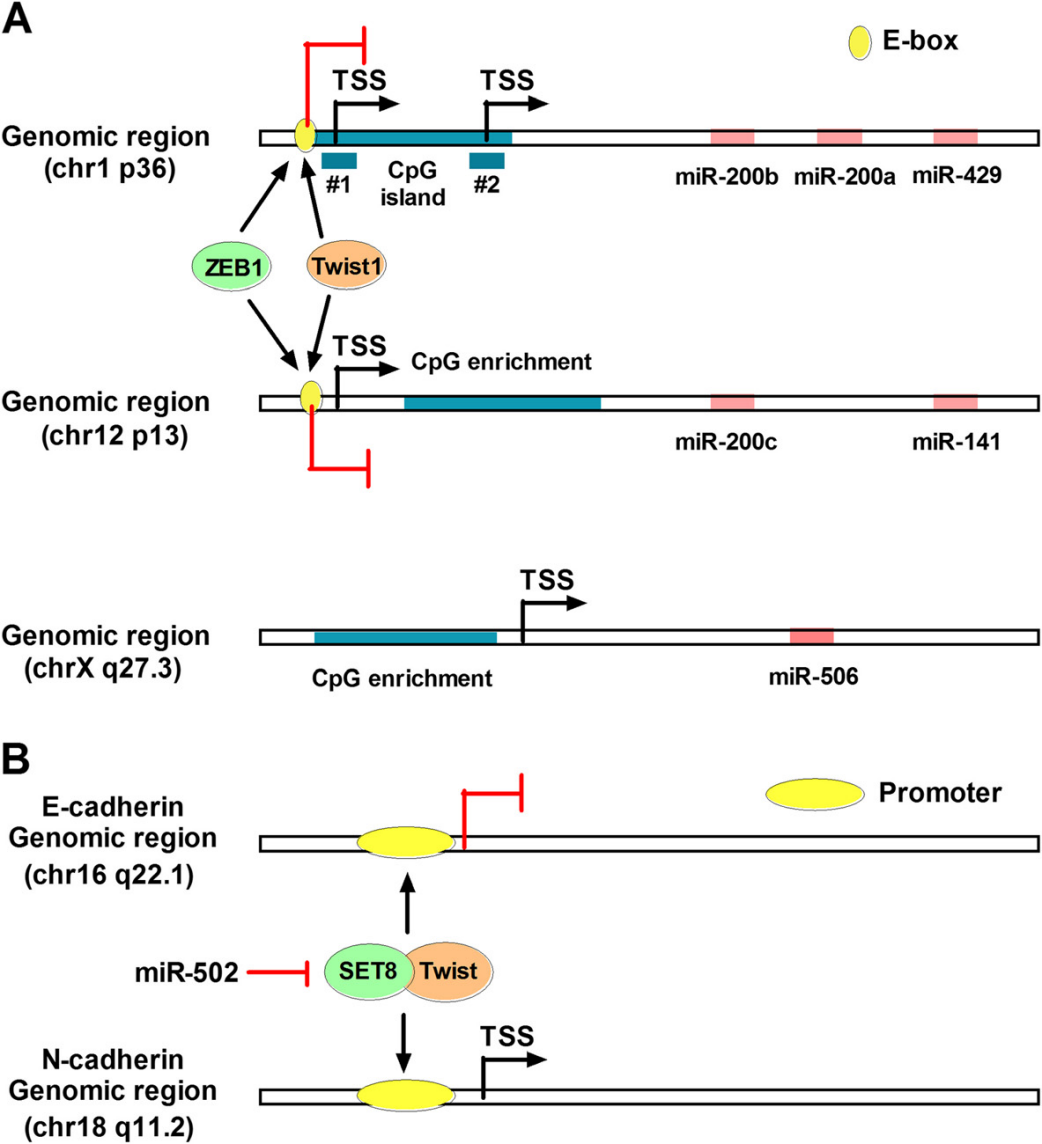
**MiR-200 and miR-506 DNA methylation genomic loci and promoters of E- and N-cadherin. A**. Graphical depiction of the miR-200b ~ 429 and miR-200c ~ 141 genomic loci, with putative transcription start sites (TSS) indicated by arrows. ZEB1 and Twist1 bound the E-box consensus in the promoters proximal to the putative miR-200 TSS and repressed miR-200 expression. The genomic position of miR-506 and five candidate methylation-regulated positions are also shown. **B**. SET8 interacted with Twist to regulate E-cadherin or N-cadherin promoter. MiR-502 suppressed SET8 directly and promoted E-cadherin expression.

The miR-200 family consists of five members in two clusters: miR-200b ~ 200a ~ 429 and miR-200c ~ 141. Studies have illustrated DNA methylation in two regions (#1 and #2) of a 2.5-kb large CpG island that is 2 kb upstream in miR-200b ~ 429 and in smaller CpG-enriched regions associated with miR-200c ~ 141. These regions can be demethylated by 5-Aza-20-deoxycytidine and the histone deacetylase inhibitor trichostatin A [56]. Aberrant DNA methylation of the CpG island or the CpG-enriched regions is closely linked to miR-200 inappropriate silencing in cancer cells [57]. Other factors may also be involved in miR-200 repression, such as ZEB1 and Twist1. A recent study showed that induction of ZEB1 and ZEB2 increased the methylation of miR-200 promoters [58]. Twist1 was also reported to directly associate with miR-200 promoters as a transcriptional repressor of miR-200 [56] (Figure 3A).

### MiRNAs other than miR-200 inhibit EMT by targeting ZEB1 and ZEB2

In addition to miR-200 family members, other miRNAs have been identified that regulate EMT by directly targeting ZEB1 and ZEB2. For example, miR-130b [59], miR-150 [60], and miR-655 [61] inhibit EMT by directly targeting ZEB1. Ectopic expression of miR-192 and miR-215 increased E-cadherin levels by targeting ZEB2 [31]. MiR-205, which is induced by p63, was reported to inhibit EMT by targeting ZEB1 and ZEB2 [35] in breast cancer [62] and prostate cancer [63]. MiR-153 is a novel regulator of EMT that targets ZEB2 and SNAI1 [64].

### MiRNAs that regulate SNAI1 and SNAI2

MiR-34 inhibits EMT by directly targeting SNAI1 [65]. Moreover, SNAI1 can repress transcription of miR-34 genes, resulting in a SNAI1/miR-34 feedback loop that is analogous to the reciprocal ZEB/miR-200 feedback loop [66]. MiR-34 targets a set of highly conserved sites in the 3′ untranslated region (UTR) of Wnt and EMT genes, specifically *WNT1*, *WNT3*, *LRP6*, *AXIN2*, *β-catenin*, and *LEF1*, resulting in suppression of TCF/LEF transcriptional activity and the EMT process [67].

MiR-203 was found to be repressed by SNAI1 during SNAI1-induced EMT in MCF7 breast cancer cells. Meanwhile, miR-203 repressed endogenous SNAI1, forming a double-negative miR203/SNAI1 feedback loop [68]. miR-203 targeted SNAI2 [69], and SNAI2 directly bound to the miR-203 promoter to inhibit its transcription. Therefore, miR-203 also formed a double-negative feedback loop with SNAI2 in which each inhibited the other’s expression, thereby controlling EMT [70]. In another double-feedback loop, miR-200 and SNAI2 regulate EMT. While SNAI2 is targeted by miR-200, SNAI2 directly binds E-boxes in the miR-200a/b promoter regions and represses miR-200a/b transcription. Therefore, SNAI2 and miR-200 act in a self-reinforcing regulatory loop that leads to amplification of EMT [71].

**Figure 4:**
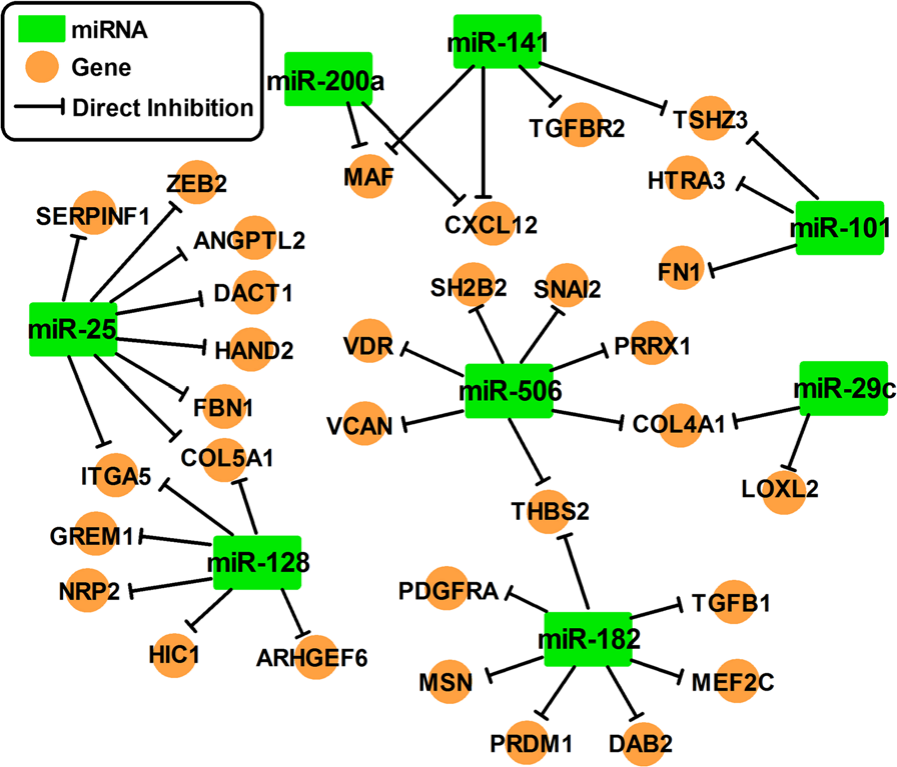
**MiRNA-gene network involved in EMT.** The miRNA-gene network shows the eight key miRNA and EMT signature genes that are predicted to be regulated. This figure is modified from one in a Cancer Cell journal paper [72].

The results of a recent report suggest that miR-506 is a novel microRNA that inhibits EMT [72]. Integrated genomic analyses revealed a miRNA-regulatory network that is involved in EMT in serous ovarian cancer (Figure 4). MiR-506 augmented E-cadherin expression, inhibited cell migration and invasion, and prevented TGFβ-induced EMT by targeting SNAI2. MiR-506 expression is downregulated in an integrated mesenchymal subtype of serous ovarian cancer through methylation of CpG sites on the miR-506 promoter (Figure 3A). The nanoparticle delivery of miR-506 in orthotopic ovarian cancer mouse models led to E-cadherin induction and reduced tumor growth [72].

Several other miRNAs also target SNAI1 and SNAI2, such as miR-182 [69], miR-30 [73], miR-1 [71], and miR-29b [74] (Figure 2).

### MiRNAs that regulate Twist1 and Twist2

The transcription factor Twist is a highly conserved basic helix-loop-helix transcription factor that promotes EMT and tumor metastasis. Apart from let-7d [75], miR-29b [74], and miR-214 [76], miR-580 was also reported to act as a negative regulator of Twist1 that induces EMT in breast cancer [77]. MiR-675 similarly directly downregulates Twist1 expression, leading to EMT [78] (Figure 2).

The results of a recent study showed that SET8 promotes EMT and enhances the invasive potential of breast cancer cells in vitro and in vivo by interacting with Twist. SET8 interacts with Twist to regulate the E-cadherin or N-cadherin promoter [79]. Fengju Song *et al.* identified a single-nucleotide polymorphism within the miR-502 seed-binding region in the 3′-UTR of the *SET8* genethat modulates SET8 expression [80]. Thus, miR-502 may suppress EMT by inhibiting SET8 (Figure 3B).

### MiRNAs directly regulate E-cadherin

Both miR-9 [81] and miR-23a [82] directly target E-cadherin, leading to increased cell motility and invasiveness (Figure 2). As miR-9 can be regulated by c-Myc and Prospero homeobox 1, overexpression of both [83,84] led to EMT; it also resulted in a significant decrease in E-cadherin and increase in vimentin through the upregulation of miR-9. In addition, miR-9 promoted EMT and metastasis by directly regulating KLF17 expression [85].

### MiRNAs regulate vimentin and fibronectin

MiR-506 was reported to inhibit TGFβ-induced EMT by directly targeting vimentin in a human breast cancer cell line [86]. MiR-30 was reported to suppress the migratory ability and invasiveness of breast cancer cell lines by directly targeting vimentin [87]. Furthermore, MiR-17-3p [88], as well as miR-124 and miR-203 [89], repressed vimentin expression by targeting its 3′UTR. miR-138 suppressed cell migration and invasion by directly targeting vimentin in renal cell carcinoma [90] and squamous cell carcinoma cells [91].

The results of another report suggested that miRNA-200b suppresses TGF-β1-induced EMT by directly targeting the 3′UTR of fibronectin [92]. Similarly, miR-17 resulted in decreased cell adhesion and migration by directly targeting fibronectin [93].

Since miRNAs play important roles in EMT and cancer metastasis, there is growing interest in using them in therapeutic applications [94]. Downregulation of the miRNAs that promote cancer progression may provide effective therapeutics for patients by using specific oligomers, called antagomirs that compete with the target mRNA to bind to miRNA. Krutzfeldt *et al.* found that antagomirs are powerful tools to silence specific miRNA *in vivo* and may represent a therapeutic strategy for silencing miRNAs in disease [95]. Meanwhile, the restoration of tumor-suppressive miRNA in tumors by external delivery may serve as a promising therapeutic option [96]. A report suggested that miR-200b and miR-200c were significantly associated with survival in gastric cancer patients; miR-200b suppressed ZEB1, augmented E-cadherin, inhibited cell migration, and suppressed tumor growth in a mouse model [97]. Furthermore, delivery of miR-200 members into the tumor endothelium resulted in marked reductions in metastasis and angiogenesis [98].

### Conclusions and future directions

A plethora of miRNAs, including miR-200 family members and miR-506, have been found to directly regulate the expression of the target genes that are known to play critical roles in EMT regulation (Figure 4). As shown in Table 1, aside from regulating the signaling pathways and transcriptional factors described above, miRNAs regulate other genes to modulate EMT in various cancer types.

**Table 1:** MiRNAs that regulate EMT and their targets in different cancer types

**Cancer type**	**miRNA**	**Target**	**Reference**
Breast	miR-24	Net1A	[99]
	miR-29a	TTP	[100]
	miR-106b-25	SMAD7	[101]
	miR-221/222	TRPS1	[102]
	miR-374a	WIF1, PTEN, WNT5A	[103]
	miR-375	MTDH	[104]
	miR-448	SATB1	[105]
	miR-506	Vimentin, SNAI2, CD151	[86]
	miR-661	Nectin-1, StarD10	[106]
GC	miR-7	IGF1R	[20]
	miR-27	APC	[107]
	miR-106b-25	SMAD7	[101]
	miR-197	p120 catenin	[108]
HCC	miR-21	PTEN, hSulf-1	[109]
	miR-194	BMI-1	[110]
	miR-490-3p	ERGIC3	[111]
	miR-491	MMP-9	[112]
	miR-612	AKT2	[113]
HNSCC	miR-138	Vimentin, ZEB2, EZH2	[91]
LAD	Let-7c	Bcl-xl	[114]
Liver	miR-216a/217	PTEN, SMAD7	[115]
Lung	miR-365	HMGA2	[116]
Melanoma	miR-137	CtBP1	[117]
NSCLC	miR-134	FOXM1	[118]
	miR-149	FOXM1	[119]
Ovarian	miR-187	Dab2	[120]
Pancreatic	miR-126	ADAM9	[121]

*Abbreviations:Net1A* Neuroepithelial cell transforming 1, *TTP* Tristetraprolin, *TRPS1* Trichorh inophalangeal 1, *WIF1* Wnt inhibitory factor-1, *MTDH* Metadherin, *SATB1* Special AT-rich sequence-binding protein-1, *GC* Gastric cancer, *IGF1R* Insulin-like growth factor-1 receptor, *APC* Adenomatous polyposis coli, *HCC* Hepatocellular carcinoma, *hSulf-1* Human sulfatase-1, *BMI-1*, B lymphoma mouse Moloney leukemia virus insertion region 1, *ERGIC3* Endoplasmic reticulum-Golgi intermediate compartment protein 3, *HNSCC* Head and neck squamous cell carcinoma, *LAD* Lung adenocarcinoma, *CtBP1* Carboxyl-terminal binding protein 1, *NSCLC* Non-small cell lung cancer, *FOXM1* Forkhead box M1, *Dab2* Disabled homolog-2, *ADAM9* Disintegrin and metalloproteinase domain-containing protein 9.

Targeting EMT and MET may provide effective therapeutics for cancer. However, therapeutic intervention may be complex because EMT occurs at an early stage of metastasis and MET occurs at later stages. MiRNAs that inhibit EMT, such as miR-141, were found in the circulation of patients with metastatic colon cancer, and high levels of plasma miR-141 were predictive of poor survival [122]. As miR-141 may promote MET, it is conceivable that miR-141 promotes tumor growth at distant sites at a late stage of metastasis, a theory that warrants further investigation. A recent report by Oscar *et al.* provided supporting evidence that MET is essential for the colonization and metastasis of differentiated carcinomas because of EMT-associated growth arrest [123]. Jeff *et al.* also demonstrated that activation of EMT promotes local tumor invasion, intravasation, and extravasation of the systemic circulation; MET is essential for establishing macrometastases [124]. As EMT is associated with decreased cell proliferation and MET promotes metastatic growth, it is still unknown whether EMT inhibition is a logical approach to preventing metastasis.

Most studies have shown that, as post-transcriptional regulators, lncRNA and miRNA play important roles in EMT and are important markers and tools in cancer diagnosis, prognosis, and therapeutics. However, lncRNA and miRNA have multiple targets that are involved in multiple different physiological processes; therefore, the role of therapeutics that target lncRNA or miRNA should be validated *in vi*vo to determine their overall physiological effect.

## Competing interests

The authors declare that they have no competing interests.

## Authors’ contributions

All authors contributed to discussing and writing this manuscript. All authors have read and approved the final manuscript.

## References

[B1] Thiery JP Sleeman JP Complex networks orchestrate epithelial-mesenchymal transitions Nat Rev Mol Cell Biol 2006 7 131 142 10.1038/nrm1835 16493418

[B2] Chang CC Hsu WH Wang CC Chou CH Kuo MY Lin BR Chen ST Tai SK Kuo ML Yang MH Connective tissue growth factor activates pluripotency genes and mesenchymal-epithelial transition in head and neck cancer cells Cancer Res 2013 73 4147 4157 10.1158/0008-5472.CAN-12-4085 23687336

[B3] Wu CY Tsai YP Wu MZ Teng SC Wu KJ Epigenetic reprogramming and post-transcriptional regulation during the epithelial-mesenchymal transition Trends Genet 2012 28 454 463 10.1016/j.tig.2012.05.005 22717049

[B4] Lamouille S Subramanyam D Blelloch R Derynck R Regulation of epithelial-mesenchymal and mesenchymal-epithelial transitions by microRNAs Curr Opin Cell Biol 2013 25 200 207 10.1016/j.ceb.2013.01.008 23434068 PMC4240277

[B5] Yu Z Pestell TG Lisanti MP Pestell RG Cancer stem cells Int J Biochem Cell Biol 2012 44 2144 2151 10.1016/j.biocel.2012.08.022 22981632 PMC3496019

[B6] Mendez MG Kojima S Goldman RD Vimentin induces changes in cell shape, motility, and adhesion during the epithelial to mesenchymal transition FASEB J 2010 2010 24 10.1096/fj.09-151639 PMC2874471 20097873

[B7] Yang J Eddy JA Pan Y Hategan A Tabus I Wang Y Cogdell D Price ND Pollock RE Lazar AJ Hunt KK Trent JC Zhang W Integrated proteomics and genomics analysis reveals a novel mesenchymal to epithelial reverting transition in leiomyosarcoma through regulation of slug Mol Cell Proteomics 2010 9 2405 2413 10.1074/mcp.M110.000240 20651304 PMC2984227

[B8] Huang T Alvarez A Hu B Cheng SY Noncoding RNAs in cancer and cancer stem cells Chin J Cancer 2013 32 582 593 10.5732/cjc.013.10170 24206916 PMC3845549

[B9] Zhao Y Guo Q Chen J Hu J Wang S Sun Y Role of long non-coding RNA HULC in cell proliferation, apoptosis and tumor metastasis of gastric cancer: a clinical and in vitro investigation Oncol Rep 2014 31 358 364 24247585 10.3892/or.2013.2850

[B10] Xu ZY Yu QM Du YA Yang LT Dong RZ Huang L Yu PF Cheng XD Knockdown of long non-coding RNA HOTAIR suppresses tumor invasion and reverses epithelial-mesenchymal transition in gastric cancer Int J Biol Sci 2013 9 587 597 10.7150/ijbs.6339 23847441 PMC3708039

[B11] Ying L Chen Q Wang Y Zhou Z Huang Y Qiu F Upregulated MALAT-1 contributes to bladder cancer cell migration by inducing epithelial-to-mesenchymal transition Mol Biosyst 2012 8 2289 2294 10.1039/c2mb25070e 22722759

[B12] Luo M Li Z Wang W Zeng Y Liu Z Qiu J Long non-coding RNA H19 increases bladder cancer metastasis by associating with EZH2 and inhibiting E-cadherin expression Cancer Lett 2013 333 213 221 10.1016/j.canlet.2013.01.033 23354591

[B13] Gupta RA Shah N Wang KC Kim J Horlings HM Wong DJ Tsai MC Hung T Argani P Rinn JL Wang Y Brzoska P Kong B Li R West RB van de Vijver MJ Sukumar S Chang HY Long non-coding RNA HOTAIR reprograms chromatin state to promote cancer metastasis Nature 2010 464 1071 1076 10.1038/nature08975 20393566 PMC3049919

[B14] Hu P Yang J Hou Y Zhang H Zeng Z Zhao L Yu T Tang X Tu G Cui X Liu M LncRNA expression signatures of twist-induced epithelial-to-mesenchymal transition in MCF10A cells Cell Signal 2014 26 83 93 10.1016/j.cellsig.2013.10.001 24113349

[B15] Li CH Chen Y Targeting long non-coding RNAs in cancers: progress and prospects Int J Biochem Cell Biol 2013 45 1895 1910 10.1016/j.biocel.2013.05.030 23748105

[B16] Hassan O Ahmad A Sethi S Sarkar FH Recent updates on the role of microRNAs in prostate cancer J Hematol Oncol 2012 5 9 10.1186/1756-8722-5-9 22417299 PMC3313897

[B17] Budhu A Ji J Wang XW The clinical potential of microRNAs J Hematol Oncol 2010 3 37 10.1186/1756-8722-3-37 20925959 PMC2958878

[B18] Musumeci M Coppola V Addario A Patrizii M Maugeri-Sacca M Memeo L Colarossi C Francescangeli F Biffoni M Collura D Giacobbe A D’Urso L Falchi M Venneri MA Muto G De Maria R Bonci D Control of tumor and microenvironment cross-talk by miR-15a and miR-16 in prostate cancer Oncogene 2011 30 4231 4242 10.1038/onc.2011.140 21532615

[B19] Tan S Li R Ding K Lobie PE Zhu T miR-198 inhibits migration and invasion of hepatocellular carcinoma cells by targeting the HGF/c-MET pathway FEBS Lett 2011 585 2229 2234 10.1016/j.febslet.2011.05.042 21658389

[B20] Zhao X Dou W He L Liang S Tie J Liu C Li T Lu Y Mo P Shi Y Wu K Nie Y Fan D MicroRNA-7 functions as an anti-metastatic microRNA in gastric cancer by targeting insulin-like growth factor-1 receptor Oncogene 2013 32 1363 1372 10.1038/onc.2012.156 22614005

[B21] Guttilla IK Adams BD White BA ERalpha, microRNAs, and the epithelial-mesenchymal transition in breast cancer Trends Endocrinol Metab 2012 23 73 82 10.1016/j.tem.2011.12.001 22257677

[B22] Du R Sun W Xia L Zhao A Yu Y Zhao L Wang H Huang C Sun S Hypoxia-induced down-regulation of microRNA-34a promotes EMT by targeting the Notch signaling pathway in tubular epithelial cells PLoS One 2012 7 e30771 10.1371/journal.pone.0030771 22363487 PMC3281867

[B23] Brabletz S Bajdak K Meidhof S Burk U Niedermann G Firat E Wellner U Dimmler A Faller G Schubert J Brabletz T The ZEB1/miR-200 feedback loop controls Notch signalling in cancer cells EMBO J 2011 30 770 782 10.1038/emboj.2010.349 21224848 PMC3041948

[B24] Saydam O Shen Y Wurdinger T Senol O Boke E James MF Tannous BA Stemmer-Rachamimov AO Yi M Stephens RM Fraefel C Gusella JF Krichevsky AM Breakefield XO Downregulated microRNA-200a in meningiomas promotes tumor growth by reducing E-cadherin and activating the Wnt/beta-catenin signaling pathway Mol Cell Biol 2009 29 5923 5940 10.1128/MCB.00332-09 19703993 PMC2772747

[B25] Gill BJ Gibbons DL Roudsari LC Saik JE Rizvi ZH Roybal JD Kurie JM West JL A synthetic matrix with independently tunable biochemistry and mechanical properties to study epithelial morphogenesis and EMT in a lung adenocarcinoma model Cancer Res 2012 72 6013 6023 10.1158/0008-5472.CAN-12-0895 22952217 PMC3632398

[B26] Xu Q Wang L Li H Han Q Li J Qu X Huang S Zhao RC Mesenchymal stem cells play a potential role in regulating the establishment and maintenance of epithelial-mesenchymal transition in MCF7 human breast cancer cells by paracrine and induced autocrine TGF-beta Int J Oncol 2012 41 959 968 22766682 10.3892/ijo.2012.1541

[B27] Xiong M Jiang L Zhou Y Qiu W Fang L Tan R Wen P Yang J The miR-200 family regulates TGF-beta1-induced renal tubular epithelial to mesenchymal transition through Smad pathway by targeting ZEB1 and ZEB2 expression Am J Physiol Renal Physiol 2012 302 F369 F379 10.1152/ajprenal.00268.2011 22012804

[B28] Turcatel G Rubin N El-Hashash A Warburton D MIR-99a and MIR-99b modulate TGF-beta induced epithelial to mesenchymal plasticity in normal murine mammary gland cells PLoS One 2012 7 e31032 10.1371/journal.pone.0031032 22299047 PMC3267767

[B29] Su A He S Tian B Hu W Zhang Z MicroRNA-221 mediates the effects of PDGF-BB on migration, proliferation, and the epithelial-mesenchymal transition in pancreatic cancer cells PLoS One 2013 8 e71309 10.1371/journal.pone.0071309 23967190 PMC3742757

[B30] Hong JP Li XM Li MX Zheng FL VEGF suppresses epithelial-mesenchymal transition by inhibiting the expression of Smad3 and miR192, a Smad3-dependent microRNA Int J Mol Med 2013 31 1436 1442 23588932 10.3892/ijmm.2013.1337

[B31] Wang B Herman-Edelstein M Koh P Burns W Jandeleit-Dahm K Watson A Saleem M Goodall GJ Twigg SM Cooper ME Kanthatidis P E-cadherin expression is regulated by miR-192/215 by a mechanism that is independent of the profibrotic effects of transforming growth factor-beta Diabetes 2010 59 1794 1802 10.2337/db09-1736 20393144 PMC2889781

[B32] Zhang Z Liu ZB Ren WM Ye XG Zhang YY The miR-200 family regulates the epithelial-mesenchymal transition induced by EGF/EGFR in anaplastic thyroid cancer cells Int J Mol Med 2012 30 856 862 22797360 10.3892/ijmm.2012.1059

[B33] Lei C Wang Y Huang Y Yu H Wu L Huang L Up-regulated miR155 reverses the epithelial-mesenchymal transition induced by EGF and increases chemo-sensitivity to cisplatin in human Caski cervical cancer cells PLoS One 2012 7 e52310 10.1371/journal.pone.0052310 23284982 PMC3527539

[B34] Mongroo PS Rustgi AK The role of the miR-200 family in epithelial-mesenchymal transition Cancer Biol Ther 2010 10 219 222 10.4161/cbt.10.3.12548 20592490 PMC3040834

[B35] Gregory PA Bert AG Paterson EL Barry SC Tsykin A Farshid G Vadas MA Khew-Goodall Y Goodall GJ The miR-200 family and miR-205 regulate epithelial to mesenchymal transition by targeting ZEB1 and SIP1 Nat Cell Biol 2008 10 593 601 10.1038/ncb1722 18376396

[B36] Burk U Schubert J Wellner U Schmalhofer O Vincan E Spaderna S Brabletz T A reciprocal repression between ZEB1 and members of the miR-200 family promotes EMT and invasion in cancer cells EMBO Rep 2008 9 582 589 10.1038/embor.2008.74 18483486 PMC2396950

[B37] Wellner U Schubert J Burk UC Schmalhofer O Zhu F Sonntag A Waldvogel B Vannier C Darling D zur Hausen A Brunton VG Morton J Sansom O Schuler J Stemmler MP Herzberger C Hopt U Keck T Brabletz S Brabletz T Darling D zur Hausen A Brunton VG Morton J Sansom O Schuler J Stemmler MP Herzberger C Hopt U Keck T Brabletz S Brabletz T The EMT-activator ZEB1 promotes tumorigenicity by repressing stemness-inhibiting microRNAs Nat Cell Biol 2009 11 1487 1495 10.1038/ncb1998 19935649

[B38] Bracken CP Gregory PA Kolesnikoff N Bert AG Wang J Shannon MF Goodall GJ A double-negative feedback loop between ZEB1-SIP1 and the microRNA-200 family regulates epithelial-mesenchymal transition Cancer Res 2008 68 7846 7854 10.1158/0008-5472.CAN-08-1942 18829540

[B39] Mizuguchi Y Specht S Lunz JG 3rd Isse K Corbitt N Takizawa T Demetris AJ Cooperation of p300 and PCAF in the control of microRNA 200c/141 transcription and epithelial characteristics PLoS One 2012 7 e32449 10.1371/journal.pone.0032449 22384255 PMC3284570

[B40] Ahn SM Cha JY Kim J Kim D Trang HT Kim YM Cho YH Park D Hong S Smad3 regulates E-cadherin via miRNA-200 pathway Oncogene 2012 31 3051 3059 10.1038/onc.2011.484 22020340

[B41] Chang CJ Chao CH Xia W Yang JY Xiong Y Li CW Yu WH Rehman SK Hsu JL Lee HH Liu M Chen CT Yu D Hung MC p53 regulates epithelial-mesenchymal transition and stem cell properties through modulating miRNAs Nat Cell Biol 2011 13 317 323 10.1038/ncb2173 21336307 PMC3075845

[B42] Schubert J Brabletz T p53 Spreads out further: suppression of EMT and stemness by activating miR-200c expression Cell Res 2011 21 705 707 10.1038/cr.2011.62 21483453 PMC3203673

[B43] Fu J Rodova M Nanta R Meeker D van Veldhuizen PJ Srivastava RK Shankar S NPV-LDE-225 (Erismodegib) inhibits epithelial mesenchymal transition and self-renewal of glioblastoma initiating cells by regulating miR-21, miR-128, and miR-200 Neuro Oncol 2013 15 691 706 10.1093/neuonc/not011 23482671 PMC3661095

[B44] Guo L Chen C Shi M Wang F Chen X Diao D Hu M Yu M Qian L Guo N Stat3-coordinated Lin-28-let-7-HMGA2 and miR-200-ZEB1 circuits initiate and maintain oncostatin M-driven epithelial-mesenchymal transition Oncogene 2013 32 5272 5282 10.1038/onc.2012.573 23318420

[B45] Kong D Li Y Wang Z Banerjee S Ahmad A Kim HR Sarkar FH miR-200 regulates PDGF-D-mediated epithelial-mesenchymal transition, adhesion, and invasion of prostate cancer cells Stem Cells 2009 27 1712 1721 10.1002/stem.101 19544444 PMC3400149

[B46] Bao B Wang Z Ali S Kong D Li Y Ahmad A Banerjee S Azmi AS Miele L Sarkar FH Notch-1 induces epithelial-mesenchymal transition consistent with cancer stem cell phenotype in pancreatic cancer cells Cancer Lett 2011 307 26 36 10.1016/j.canlet.2011.03.012 21463919 PMC3104092

[B47] Sureban SM May R Qu D Weygant N Chandrakesan P Ali N Lightfoot SA Pantazis P Rao CV Postier RG Houchen CW DCLK1 Regulates Pluripotency and Angiogenic Factors via microRNA-Dependent Mechanisms in Pancreatic Cancer PLoS One 2013 8 e73940 10.1371/journal.pone.0073940 24040120 PMC3767662

[B48] Grassian AR Lin F Barrett R Liu Y Jiang W Korpal M Astley H Gitterman D Henley T Howes R Levell J Korn JM Pagliarini R Isocitrate dehydrogenase (IDH) mutations promote a reversible ZEB1/microRNA (miR)-200-dependent epithelial-mesenchymal transition (EMT) J Biol Chem 2012 287 42180 42194 10.1074/jbc.M112.417832 23038259 PMC3516763

[B49] Martello G Rosato A Ferrari F Manfrin A Cordenonsi M Dupont S Enzo E Guzzardo V Rondina M Spruce T Parenti AR Daidone MG Bicciato S Piccolo S A MicroRNA targeting dicer for metastasis control Cell 2010 141 1195 1207 10.1016/j.cell.2010.05.017 20603000

[B50] Li BL Lu C Lu W Yang TT Qu J Hong X Wan XP miR-130b is an EMT-related microRNA that targets DICER1 for aggression in endometrial cancer Med Oncol 2013 30 484 23392577 10.1007/s12032-013-0484-0

[B51] Parker BC Zhang W Fusion genes in solid tumors: an emerging target for cancer diagnosis and treatment Chin J Cancer 2013 32 594 603 10.5732/cjc.013.10178 24206917 PMC3845546

[B52] Yoshimoto M Joshua AM Chilton-Macneill S Bayani J Selvarajah S Evans AJ Zielenska M Squire JA Three-color FISH analysis of TMPRSS2/ERG fusions in prostate cancer indicates that genomic microdeletion of chromosome 21 is associated with rearrangement Neoplasia 2006 8 465 469 10.1593/neo.06283 16820092 PMC1601467

[B53] Leshem O Madar S Kogan-Sakin I Kamer I Goldstein I Brosh R Cohen Y Jacob-Hirsch J Ehrlich M Ben-Sasson S Goldfinger N Loewenthal R Gazit E Rotter V Berger R TMPRSS2/ERG promotes epithelial to mesenchymal transition through the ZEB1/ZEB2 axis in a prostate cancer model PLoS One 2011 6 e21650 10.1371/journal.pone.0021650 21747944 PMC3128608

[B54] Hur K Toiyama Y Takahashi M Balaguer F Nagasaka T Koike J Hemmi H Koi M Boland CR Goel A MicroRNA-200c modulates epithelial-to-mesenchymal transition (EMT) in human colorectal cancer metastasis Gut 2013 62 1315 1326 10.1136/gutjnl-2011-301846 22735571 PMC3787864

[B55] Neves R Scheel C Weinhold S Honisch E Iwaniuk KM Trompeter HI Niederacher D Wernet P Santourlidis S Uhrberg M Role of DNA methylation in miR-200c/141 cluster silencing in invasive breast cancer cells BMC Res Notes 2010 3 219 10.1186/1756-0500-3-219 20682048 PMC3161370

[B56] Wiklund ED Bramsen JB Hulf T Dyrskjot L Ramanathan R Hansen TB Villadsen SB Gao S Ostenfeld MS Borre M Peter ME Orntoft TF Kjems J Clark SJ Coordinated epigenetic repression of the miR-200 family and miR-205 in invasive bladder cancer Int J Cancer 2011 128 1327 1334 10.1002/ijc.25461 20473948

[B57] Vrba L Jensen TJ Garbe JC Heimark RL Cress AE Dickinson S Stampfer MR Futscher BW Role for DNA methylation in the regulation of miR-200c and miR-141 expression in normal and cancer cells PLoS One 2010 5 e8697 10.1371/journal.pone.0008697 20084174 PMC2805718

[B58] Wang Z Zhao Y Smith E Goodall GJ Drew PA Brabletz T Yang C Reversal and prevention of arsenic-induced human bronchial epithelial cell malignant transformation by microRNA-200b Toxicol Sci 2011 121 110 122 10.1093/toxsci/kfr029 21292642 PMC3080188

[B59] Dong P Karaayvaz M Jia N Kaneuchi M Hamada J Watari H Sudo S Ju J Sakuragi N Mutant p53 gain-of-function induces epithelial-mesenchymal transition through modulation of the miR-130b-ZEB1 axis Oncogene 2013 32 3286 3295 10.1038/onc.2012.334 22847613 PMC3705163

[B60] Yokobori T Suzuki S Tanaka N Inose T Sohda M Sano A Sakai M Nakajima M Miyazaki T Kato H Kuwano H MiR-150 is associated with poor prognosis in esophageal squamous cell carcinoma via targeting the EMT inducer ZEB1 Cancer Sci 2013 104 48 54 10.1111/cas.12030 23013135 PMC7657108

[B61] Harazono Y Muramatsu T Endo H Uzawa N Kawano T Harada K Inazawa J Kozaki k miR-655 Is an EMT-suppressive MicroRNA targeting ZEB1 and TGFBR2 PLoS One 2013 8 e62757 10.1371/journal.pone.0062757 23690952 PMC3653886

[B62] Lee JY Park MK Park JH Lee HJ Shin DH Kang Y Lee CH Kong G Loss of the polycomb protein Mel-18 enhances the epithelial-mesenchymal transition by ZEB1 and ZEB2 expression through the downregulation of miR-205 in breast cancer Oncogene 2013 doi: 10.1038/onc.2013.53. [Epub ahead of print] 10.1038/onc.2013.53 23474752

[B63] Tucci P Agostini M Grespi F Markert EK Terrinoni A Vousden KH Muller PA Dotsch V Kehrloesser S Sayan BS Giaccone G Lowe SW Takahashi N Vandenabeele P Knight RA Levine AJ Melino G Loss of p63 and its microRNA-205 target results in enhanced cell migration and metastasis in prostate cancer Proc Natl Acad Sci U S A 2012 109 15312 15317 10.1073/pnas.1110977109 22949650 PMC3458363

[B64] Xu Q Sun Q Zhang J Yu J Chen W Zhang Z Downregulation of miR-153 contributes to epithelial-mesenchymal transition and tumor metastasis in human epithelial cancer Carcinogenesis 2013 34 539 549 10.1093/carcin/bgs374 23188671

[B65] Kim NH Kim HS Li XY Lee I Choi HS Kang SE Cha SY Ryu JK Yoon D Fearon ER Rowe RG Lee S Maher CA Weiss SJ Yook JI A p53/miRNA-34 axis regulates Snail1-dependent cancer cell epithelial-mesenchymal transition J Cell Biol 2011 195 417 433 10.1083/jcb.201103097 22024162 PMC3206336

[B66] Brabletz T MiR-34 and SNAIL: another double-negative feedback loop controlling cellular plasticity/EMT governed by p53 Cell Cycle 2012 11 215 216 10.4161/cc.11.2.18900 22214667

[B67] Cha YH Kim NH Park C Lee I Kim HS Yook JI MiRNA-34 intrinsically links p53 tumor suppressor and Wnt signaling Cell Cycle 2012 11 1273 1281 10.4161/cc.19618 22421157

[B68] Moes M le Bechec A Crespo I Laurini C Halavatyi A Vetter G Del Sol A Friederich E A novel network integrating a miRNA-203/SNAI1 feedback loop which regulates epithelial to mesenchymal transition PLoS One 2012 7 e35440 10.1371/journal.pone.0035440 22514743 PMC3325969

[B69] Qu Y Li WC Hellem MR Rostad K Popa M McCormack E Oyan AM Kalland KH Ke XS MiR-182 and miR-203 induce mesenchymal to epithelial transition and self-sufficiency of growth signals via repressing SNAI2 in prostate cells Int J Cancer 2013 133 544 555 10.1002/ijc.28056 23354685

[B70] Ding X Park SI McCauley LK Wang CY Signaling between transforming growth factor beta (TGF-beta) and transcription factor SNAI2 represses expression of microRNA miR-203 to promote epithelial-mesenchymal transition and tumor metastasis J Biol Chem 2013 288 10241 10253 10.1074/jbc.M112.443655 23447531 PMC3624408

[B71] Liu YN Yin JJ Abou-Kheir W Hynes PG Casey OM Fang L Yi M Stephens RM Seng V Sheppard-Tillman H Martin P Kelly K MiR-1 and miR-200 inhibit EMT via Slug-dependent and tumorigenesis via Slug-independent mechanisms Oncogene 2013 32 296 306 10.1038/onc.2012.58 22370643 PMC7580497

[B72] Yang D Sun Y Hu L Zheng H Ji P Pecot CV Zhao Y Reynold S Cheng H Rupaimoole R Cogdell D Nykter M Broaddus R Rodriguez-Aguayo C Lopez-Berestein G Liu J Shmulevich I Sood AK Chen K Zhang W Integrated analyses identify a master microRNA regulatory network for the mesenchymal subtype in serous ovarian cancer Cancer Cell 2013 23 186 199 10.1016/j.ccr.2012.12.020 23410973 PMC3603369

[B73] Kumarswamy R Mudduluru G Ceppi P Muppala S Kozlowski M Niklinski J Papotti M Allgayer H MicroRNA-30a inhibits epithelial-to-mesenchymal transition by targeting Snai1 and is downregulated in non-small cell lung cancer Int J Cancer 2012 130 2044 2053 10.1002/ijc.26218 21633953

[B74] Ru P Steele R Newhall P Phillips NJ Toth K Ray RB miRNA-29b suppresses prostate cancer metastasis by regulating epithelial-mesenchymal transition signaling Mol Cancer Ther 2012 11 1166 1173 10.1158/1535-7163.MCT-12-0100 22402125

[B75] Chang CJ Hsu CC Chang CH Tsai LL Chang YC Lu SW Yu CH Huang HS Wang JJ Tsai CH Chou MY Yu CC Hu FW Let-7d functions as novel regulator of epithelial-mesenchymal transition and chemoresistant property in oral cancer Oncol Rep 2011 26 1003 1010 21725603 10.3892/or.2011.1360

[B76] Li B Han Q Zhu Y Yu Y Wang J Jiang X Down-regulation of miR-214 contributes to intrahepatic cholangiocarcinoma metastasis by targeting Twist FEBS J 2012 279 2393 2398 22540680 10.1111/j.1742-4658.2012.08618.x

[B77] Nairismagi ML Vislovukh A Meng Q Kratassiouk G Beldiman C Petretich M Groisman R Fuchtbauer EM Harel-Bellan A Groisman I Translational control of TWIST1 expression in MCF-10A cell lines recapitulating breast cancer progression Oncogene 2012 31 4960 4966 10.1038/onc.2011.650 22266852

[B78] Hernandez JM Elahi A Clark CW Wang J Humphries LA Centeno B Bloom G Fuchs BC Yeatman T Shibata D miR-675 Mediates Downregulation of Twist1 and Rb in AFP-Secreting Hepatocellular Carcinoma Ann Surg Oncol 2013 Suppl 3 S625 635 23864307 10.1245/s10434-013-3106-3

[B79] Yang F Sun L Li Q Han X Lei L Zhang H Shang Y SET8 promotes epithelial-mesenchymal transition and confers TWIST dual transcriptional activities EMBO J 2012 31 110 123 10.1038/emboj.2011.364 21983900 PMC3252577

[B80] Song F Zheng H Liu B Wei S Dai H Zhang L Calin GA Hao X Wei Q Zhang W Chen K An miR-502-binding site single-nucleotide polymorphism in the 3′-untranslated region of the SET8 gene is associated with early age of breast cancer onset Clin Cancer Res 2009 15 6292 6300 10.1158/1078-0432.CCR-09-0826 19789321

[B81] Ma L Young J Prabhala H Pan E Mestdagh P Muth D Teruya-Feldstein J Reinhardt F Onder TT Valastyan S Westermann F Speleman F Vandesompele J Weinberg RA miR-9, a MYC/MYCN-activated microRNA, regulates E-cadherin and cancer metastasis Nat Cell Biol 2010 12 247 256 20173740 10.1038/ncb2024 PMC2845545

[B82] Cao M Seike M Soeno C Mizutani H Kitamura K Minegishi Y Noro R Yoshimura A Cai L Gemma A MiR-23a regulates TGF-beta-induced epithelial-mesenchymal transition by targeting E-cadherin in lung cancer cells Int J Oncol 2012 41 869 875 22752005 10.3892/ijo.2012.1535 PMC3582905

[B83] Khew-Goodall Y Goodall GJ Myc-modulated miR-9 makes more metastases Nat Cell Biol 2010 12 209 211 20173743 10.1038/ncb0310-209

[B84] Lu MH Huang CC Pan MR Chen HH Hung WC Prospero homeobox 1 promotes epithelial-mesenchymal transition in colon cancer cells by inhibiting E-cadherin via miR-9 Clin Cancer Res 2012 18 6416 6425 10.1158/1078-0432.CCR-12-0832 23045246

[B85] Sun Z Han Q Zhou N Wang S Lu S Bai C Zhao RC MicroRNA-9 enhances migration and invasion through KLF17 in hepatocellular carcinoma Mol Oncol 2013 7 884 894 10.1016/j.molonc.2013.04.007 23684102 PMC5528452

[B86] Arora H Qureshi R Park WY miR-506 regulates epithelial mesenchymal transition in breast cancer cell lines PLoS One 2013 8 e64273 10.1371/journal.pone.0064273 23717581 PMC3661463

[B87] Cheng CW Wang HW Chang CW Chu HW Chen CY Yu JC Chao JI Liu HF Ding SL Shen CY MicroRNA-30a inhibits cell migration and invasion by downregulating vimentin expression and is a potential prognostic marker in breast cancer Breast Cancer Res Treat 2012 134 1081 1093 10.1007/s10549-012-2034-4 22476851

[B88] Shan SW Fang L Shatseva T Rutnam ZJ Yang X Du W Lu WY Xuan JW Deng Z Yang BB Mature miR-17-5p and passenger miR-17-3p induce hepatocellular carcinoma by targeting PTEN, GalNT7 and vimentin in different signal pathways J Cell Sci 2013 126 1517 1530 10.1242/jcs.122895 23418359

[B89] Furuta M Kozaki KI Tanaka S Arii S Imoto I Inazawa J miR-124 and miR-203 are epigenetically silenced tumor-suppressive microRNAs in hepatocellular carcinoma Carcinogenesis 2010 31 766 776 10.1093/carcin/bgp250 19843643

[B90] Yamasaki T Seki N Yamada Y Yoshino H Hidaka H Chiyomaru T Nohata N Kinoshita T Nakagawa M Enokida H Tumor suppressive microRNA138 contributes to cell migration and invasion through its targeting of vimentin in renal cell carcinoma Int J Oncol 2012 41 805 817 22766839 10.3892/ijo.2012.1543 PMC3582944

[B91] Liu X Wang C Chen Z Jin Y Wang Y Kolokythas A Dai Y Zhao X MicroRNA-138 suppresses epithelial-mesenchymal transition in squamous cell carcinoma cell lines Biochem J 2011 440 23 31 10.1042/BJ20111006 21770894 PMC3331719

[B92] Tang O Chen XM Shen S Hahn M Pollock CA MiRNA-200b represses transforming growth factor-beta1-induced EMT and fibronectin expression in kidney proximal tubular cells Am J Physiol Renal Physiol 2013 304 F1266 1273 10.1152/ajprenal.00302.2012 23408168

[B93] Shan SW Lee DY Deng Z Shatseva T Jeyapalan Z Du WW Zhang Y Xuan JW Yee SP Siragam V Yang BB MicroRNA MiR-17 retards tissue growth and represses fibronectin expression Nat Cell Biol 2009 11 1031 1038 10.1038/ncb1917 19633662

[B94] Chai S Ma S Clinical implications of microRNAs in liver cancer stem cells Chin J Cancer 2013 32 419 426 10.5732/cjc.013.10038 23668930 PMC3845583

[B95] Krutzfeldt J Rajewsky N Braich R Rajeev KG Tuschl T Manoharan M Stoffel M Silencing of microRNAs in vivo with ‘antagomirs’ Nature 2005 438 685 689 10.1038/nature04303 16258535

[B96] Rupaimoole R Han HD Lopez-Berestein G Sood AK MicroRNA therapeutics: principles, expectations, and challenges Chin J Cancer 2011 30 368 370 10.5732/cjc.011.10186 21627858 PMC4013410

[B97] Song F Yang D Liu B Guo Y Zheng H Li L Wang T Yu J Zhao Y Niu R Liang H Winkler H Zhang W Hao X Chen K Integrated microRNA network analyses identify a poor-prognosis subtype of gastric cancer characterized by the miR-200 family Clin Cancer Res 2013 20 878 889 24352645 10.1158/1078-0432.CCR-13-1844

[B98] Pecot CV Rupaimoole R Yang D Akbani R Ivan C Lu C Wu S Han HD Shah MY Rodriguez-Aguayo C Bottsford-Miller J Liu Y Kim SB Unruh A Gonzalez-Villasana V Huang L Zand B Moreno-Smith M Mangala LS Taylor M Dalton HJ Sehgal V Wen Y Kang Y Baggerly KA Lee JS Ram PT Ravoori MK Kundra V Zhang X Tumour angiogenesis regulation by the miR-200 family Nat Commun 2013 4 2427 24018975 10.1038/ncomms3427 PMC3904438

[B99] Papadimitriou E Vasilaki E Vorvis C Iliopoulos D Moustakas A Kardassis D Stournaras C Differential regulation of the two RhoA-specific GEF isoforms Net1/Net1A by TGF-beta and miR-24: role in epithelial-to-mesenchymal transition Oncogene 2012 31 2862 2875 10.1038/onc.2011.457 21986943

[B100] Gebeshuber CA Zatloukal K Martinez J miR-29a suppresses tristetraprolin, which is a regulator of epithelial polarity and metastasis EMBO Rep 2009 10 400 405 10.1038/embor.2009.9 19247375 PMC2672883

[B101] Smith AL Iwanaga R Drasin DJ Micalizzi DS Vartuli RL Tan AC Ford HL The miR-106b-25 cluster targets Smad7, activates TGF-beta signaling, and induces EMT and tumor initiating cell characteristics downstream of Six1 in human breast cancer Oncogene 2012 31 5162 5171 10.1038/onc.2012.11 22286770 PMC3342483

[B102] Stinson S Lackner MR Adai AT Yu N Kim HJ O’Brien C Spokerke J Jhunjhunwala S Boyd Z Januario T Newman RJ Yue P Bourgon R Modrusan Z Stern HM Warming S de Sauvage FJ Amler L Yeh RF Dornan D TRPS1 targeting by miR-221/222 promotes the epithelial-to-mesenchymal transition in breast cancer Sci Signal 2011 4 ra41 10.1126/scisignal.4159ec41 21673316

[B103] Cai J Guan H Fang L Yang Y Zhu X Yuan J Wu J Li M MicroRNA-374a activates Wnt/beta-catenin signaling to promote breast cancer metastasis J Clin Invest 2013 123 566 579 23321667 10.1172/JCI65871 PMC3561816

[B104] Ward A Balwierz A Zhang JD Kublbeck M Pawitan Y Hielscher T Wiemann S Sahin O Re-expression of microRNA-375 reverses both tamoxifen resistance and accompanying EMT-like properties in breast cancer Oncogene 2013 32 1173 1182 10.1038/onc.2012.128 22508479

[B105] Li QQ Chen ZQ Cao XX Xu JD Xu JW Chen YY Wang WJ Chen Q Tang F Liu XP Xu ZD Involvement of NF-kappaB/miR-448 regulatory feedback loop in chemotherapy-induced epithelial-mesenchymal transition of breast cancer cells Cell Death Differ 2011 18 16 25 10.1038/cdd.2010.103 20798686 PMC3131865

[B106] Vetter G Saumet A Moes M Vallar L le Bechec A Laurini C Sabbah M Arar K Theillet C Lecellier CH Friederich E miR-661 expression in SNAI1-induced epithelial to mesenchymal transition contributes to breast cancer cell invasion by targeting Nectin-1 and StarD10 messengers Oncogene 2010 29 4436 4448 10.1038/onc.2010.181 20543867 PMC3506903

[B107] Zhang Z Liu S Shi R Zhao G miR-27 promotes human gastric cancer cell metastasis by inducing epithelial-to-mesenchymal transition Cancer Genet 2011 204 486 491 10.1016/j.cancergen.2011.07.004 22018270

[B108] Hamada S Satoh K Miura S Hirota M Kanno A Masamune A Kikuta K Kume K Unno J Egawa S Motoi F Unno M Shimosegawa T miR-197 induces epithelial-mesenchymal transition in pancreatic cancer cells by targeting p120 catenin J Cell Physiol 2013 228 1255 1263 10.1002/jcp.24280 23139153

[B109] Bao L Yan Y Xu C Ji W Shen S Xu G Zeng Y Sun B Qian H Chen L Wu M Su C Chen J MicroRNA-21 suppresses PTEN and hSulf-1 expression and promotes hepatocellular carcinoma progression through AKT/ERK pathways Cancer Lett 2013 337 226 236 10.1016/j.canlet.2013.05.007 23684551

[B110] Dong P Kaneuchi M Watari H Hamada J Sudo S Ju J Sakuragi N MicroRNA-194 inhibits epithelial to mesenchymal transition of endometrial cancer cells by targeting oncogene BMI-1 Mol Cancer 2011 10 99 10.1186/1476-4598-10-99 21851624 PMC3173388

[B111] Zhang LY Liu M Li X Tang H miR-490-3p modulates cell growth and epithelial to mesenchymal transition of hepatocellular carcinoma cells by targeting endoplasmic reticulum-Golgi intermediate compartment protein 3 (ERGIC3) J Biol Chem 2013 288 4035 4047 10.1074/jbc.M112.410506 23212913 PMC3567655

[B112] Zhou Y Li Y Ye J Jiang R Yan H Yang X Liu Q Zhang J MicroRNA-491 is involved in metastasis of hepatocellular carcinoma by inhibitions of matrix metalloproteinase and epithelial to mesenchymal transition Liver Int 2013 33 1271 1280 10.1111/liv.12190 23725476

[B113] Tao ZH Wan JL Zeng LY Xie L Sun HC Qin LX Wang L Zhou J Ren ZG Li YX Fan J Wu WZ miR-612 suppresses the invasive-metastatic cascade in hepatocellular carcinoma J Exp Med 2013 210 789 803 10.1084/jem.20120153 23478189 PMC3620363

[B114] Cui SY Huang JY Chen YT Song HZ Feng B Huang GC Wang R Chen LB De W Let-7c governs the acquisition of chemo- or radioresistance and epithelial-to-mesenchymal transition phenotypes in docetaxel-resistant lung adenocarcinoma Mol Cancer Res 2013 11 699 713 10.1158/1541-7786.MCR-13-0019-T 23562878

[B115] Xia H Ooi LL Hui KM MicroRNA-216a/217-induced epithelial-mesenchymal transition targets PTEN and SMAD7 to promote drug resistance and recurrence of liver cancer Hepatology 2013 58 629 641 10.1002/hep.26369 23471579

[B116] Qi J Rice SJ Salzberg AC Runkle EA Liao J Zander DS Mu D MiR-365 regulates lung cancer and developmental gene thyroid transcription factor 1 Cell Cycle 2012 11 177 186 10.4161/cc.11.1.18576 22185756 PMC3272236

[B117] Luo C Tetteh PW Merz PR Dickes E Abukiwan A Hotz-Wagenblatt A Holland-Cunz S Sinnberg T Schittek B Schadendorf D Diederichs S Eichmuller SB miR-137 inhibits the invasion of melanoma cells through downregulation of multiple oncogenic target genes J Invest Dermatol 2013 133 768 775 10.1038/jid.2012.357 23151846

[B118] Li J Wang Y Luo J Fu Z Ying J Yu Y Yu W miR-134 inhibits epithelial to mesenchymal transition by targeting FOXM1 in non-small cell lung cancer cells FEBS Lett 2012 586 3761 3765 10.1016/j.febslet.2012.09.016 23010597

[B119] Ke Y Zhao W Xiong J Cao R miR-149 inhibits Non-small-cell lung cancer cells EMT by targeting FOXM1 Biochem Res Int 2013 2013 506731 23762558 10.1155/2013/506731 PMC3671264

[B120] Chao A Lin CY Lee YS Tsai CL Wei PC Hsueh S Wu TI Tsai CN Wang CJ Chao AS Wang TH Lai CH Regulation of ovarian cancer progression by microRNA-187 through targeting Disabled homolog-2 Oncogene 2012 31 764 775 10.1038/onc.2011.269 21725366

[B121] Hamada S Satoh K Fujibuchi W Hirota M Kanno A Unno J Masamune A Kikuta K Kume K Shimosegawa T MiR-126 acts as a tumor suppressor in pancreatic cancer cells via the regulation of ADAM9 Mol Cancer Res 2012 10 3 10 10.1158/1541-7786.MCR-11-0272 22064652

[B122] Cheng H Zhang L Cogdell DE Zheng H Schetter AJ Nykter M Harris CC Chen K Hamilton SR Zhang W Circulating plasma MiR-141 is a novel biomarker for metastatic colon cancer and predicts poor prognosis PLoS One 2011 6 e17745 10.1371/journal.pone.0017745 21445232 PMC3060165

[B123] Ocana OH Corcoles R Fabra A Moreno-Bueno G Acloque H Vega S Barrallo-Gimeno A Cano A Nieto MA Metastatic colonization requires the repression of the epithelial-mesenchymal transition inducer Prrx1 Cancer Cell 2012 22 709 724 10.1016/j.ccr.2012.10.012 23201163

[B124] Tsai JH Donaher JL Murphy DA Chau S Yang J Spatiotemporal regulation of epithelial-mesenchymal transition is essential for squamous cell carcinoma metastasis Cancer Cell 2012 22 725 736 10.1016/j.ccr.2012.09.022 23201165 PMC3522773

